# Microbiome of highly polluted coal mine drainage from Onyeama, Nigeria, and its potential for sequestrating toxic heavy metals

**DOI:** 10.1038/s41598-021-96899-z

**Published:** 2021-09-01

**Authors:** Ganiyu Oladunjoye Oyetibo, Joy Aimiede Enahoro, Chimuanya Amarachi Ikwubuzo, Chiamaka Shileakanwa Ukwuoma

**Affiliations:** 1 Department of Microbiology, Faculty of Science, University of Lagos, Akoka, Yaba, 101017 Lagos Nigeria; 2 Institute of Maritime, University of Lagos, Akoka, Yaba, 101017 Lagos Nigeria

**Keywords:** Biotechnology, Ecology, Microbiology, Molecular biology, Ecology, Environmental sciences

## Abstract

Drains from coal mines remain a worrisome point-source of toxic metal/metalloid pollutions to the surface- and ground-waters worldwide, requiring sustainable remediation strategies. Understanding the microbial community subtleties through microbiome and geochemical data can provide valuable information on the problem. Furthermore, the autochthonous microorganisms offer a potential means to remediate such contamination. The drains from Onyeama coal mine in Nigeria contained characteristic sulphates (313.0 ± 15.9 mg l^−1^), carbonate (253.0 ± 22.4 mg l^−1^), and nitrate (86.6 ± 41.0 mg l^−1^), having extreme tendencies to enrich receiving environments with extremely high pollution load index (3110 ± 942) for toxic metals/metalloid. The drains exerted severe degree of toxic metals/metalloid contamination (Degree of contamination: 3,400,000 ± 240,000) and consequent astronomically high ecological risks in the order: Lead > Cadmium > Arsenic > Nickel > Cobalt > Iron > Chromium. The microbiome of the drains revealed the dominance of Proteobacteria (50.8%) and Bacteroidetes (18.9%) among the bacterial community, whereas Ascomycota (60.8%) and Ciliophora (12.6%) dominated the eukaryotic community. A consortium of 7 autochthonous bacterial taxa exhibited excellent urease activities (≥ 253 µmol urea min^−1^) with subsequent stemming of acidic pH to > 8.2 and sequestration of toxic metals (approx. 100% efficiency) as precipitates (15.6 ± 0.92 mg ml^−1^). The drain is a point source for metals/metalloid pollution, and its bioremediation is achievable with the bacteria consortium.

## Introduction

Coal mining is one of the foremost global geogenic and anthropogenic sources of heavy metals (HMs) pollution through acid mine drainages (AMD). AMD is distinctive fluvial wastewater originating from the natural oxidation of sulphide minerals of mining wastes at mine sites. An insidious feature of AMD is that its sources may remain active for decades or even centuries after mine closure, continuously creating environmentally hazardous scenarios. These sites exist globally in many places that have sometimes, or still, depended on coal for energy generation. Not less than 19,300 km of rivers and 72,000 ha of lakes and reservoirs worldwide are severely affected by mining effluents^1^. AMD pollution is a major problem encountered in the supply of potable water to residents of Enugu and other mining areas in Nigeria is^2^.

The environmental stressors contained in AMD include mobile toxic HMs; SO_4_^2−^, and H^+^ that is responsible for the acidic pH; and relatively low (< 20 mg l^−1^) concentrations of dissolved organic matter^3^. AMD causes surface- and ground-water to become acidic (pH as low as 2); triggers eutrophication via sulphate enrichment, and makes metal load become a menace that often hampers agriculture and endangers aquatic life. The metals/metalloids either wield direct toxicity on the ecosystems^4,5^, or their precipitates cloak the water-beds of receiving hydrosphere^6,7^. The dissolved toxic metals/metalloids in coal AMD include iron (Fe), aluminium (Al), zinc (Zn), copper (Cu), cadmium (Cd), mercury (Hg), lead (Pb), arsenic (As) etc. Environmental pollution with coal AMD exacerbate public health hazards via metals/metalloids toxicity, leading to fatality or permanent deformity of humans and unborn babies in many cases^5,8^. Cases of metal poisoning of rural dwellers emanating from artisanal mining and consequent AMD pollution of surface waters occur unabated, but rarely reported, in countries with low incomes (personal observation of Nigeria mining villages).

The impact of AMD on receiving environments has been the focus of extensive restoration efforts. The goals of such efforts involve reducing metal loads and enhancing biotic structure (diversity and abundance)^9^. Remediation of AMD via abiotic stratagems and wetland construction has been suggested with some limitations^10–12^. Therefore, the activities of autochthonous microorganisms are the best eco-friendly option for decommissioning AMD stressors^9,12,13^. In conjunction with oligotrophic heterotrophs (such as *Acidocella* and *Thermoplasma*), the early microbial colonizers indirectly ameliorate HMs toxicity via syntrophic commensal associations with iron- and sulphur-oxidizers^3^. Thus, they utilize organic compounds (cell exudates and lysates) originating from the autotrophic primary producers, thereby essentially “detoxifying” the environment for the later groups of microorganisms^14,15^. Sulphate reducing bacteria (SRB) belonging to the genus *Desulfosporosinus* and *Desulfitobacterium,* and phyla of Firmicutes and Actinobacteria have been detected in mine-lakes reversing the chemical reactions that formed AMD, attenuating toxic metals/metalloids concentrations by sulphide precipitation, and raising the pH of the acidic water^3,6,10^.

Attempt to ameliorate the harmful environmental effects of coal AMD necessitated evaluating geochemical hazards and characterising the microbial structure of AMD stream from coal mine systems in the present study. The search for autochthonous microorganisms, particularly those producing urease activities^16^, is postulated as a novel application in the bioremediation of AMD. Whereby, urease production by the selected microbes would hydrolyse urea into ammonia and carbamate (CO(NH_2_)_2_ + H_2_O → NH_2_COOH + NH_3_), which subsequently releases ammonia and carbonic acid (NH_2_COOH + H_2_O → NH_3_ + H_2_CO_3_) that variously stem acidic and toxic metals impacts in the biosphere^7,17–19^. The need to provide insight into taxa that are pivotal to the ecophysiology of stemming AMD stream is apt for knowledge-based bioremediation of AMD from coal mining. Therefore, integration of culture-independent microbiomes, through sequencing of small subunits (SSU) DNA barcodes, with geochemical data of AMD is therefore sought in this study. This approach will decipher underline dynamics in microbial community structure of drains from a coal mine. Furthermore, culture enrichment of the AMD stream will provide information on key players involved in alleviating the metal/metalloid loads contained in the AMD.

## Results and discussion

### Geochemistry and ecotoxicology of AMD

AMD systems are an important source of metal/metalloid pollution to the receiving hydrosphere with devastating consequences on the biological drivers of affected ecosystems. Environmental menaces of AMD have not been exhaustively reported worldwide. Scanty information exists across Africa and many developing economies. The homogenised mixture of detached biofilm and AMD samples from a derelict coal mine at three sampling periods were assayed for geochemical delineation and analysed for pollution intensity against reference background geochemical values. The measured values of the physical properties and contents of selected HMs in drains from a coal mine in Nigeria were as presented in Supplementary Table A.1. Virtually all the measured parameters exceeded the permissible limits of WHO guidelines for potable water. The AMD water was acidic (pH = 3.1 ± 0.265), and contained characteristic anions that are common to AMD including dissolved sulphides (1.37 ± 0.233 mg l^−1^), sulphates (313.0 ± 15.9 mg l^−1^), carbonate (253.0 ± 22.4 mg l^−1^) and nitrate (86.6 ± 41.0 mg l^−1^) above the allowable limits of WHO. Although the acidic pH of AMD in the present study compares well with those associated with mines in Russia^14^, more extreme acidic pH values have been reported in other climes. Negative pH values of − 1.56 and − 3.6 were observed in AMD from Iberian Pyrite Belt^20^ and Richmond Mine at Iron Mountain, USA^21^, respectively. The values of physicochemical parameters associated with the AMD from Onyeama were similar to data reported for other mine wastewaters in Nigeria^22^ and elsewhere^4^. It is known that sulphide minerals, in presence of water and oxygen, oxidise to sulphate as observed in the elevated sulphate concentration (313 ± 15.9 mg l^−1^) in the present study. The low pH observed in the AMD is due to the formation of sulphuric acid from sulphate in presence of protons (H^+^). This consequently causes the leaching of metal/metalloid ions into the drains. The concentrations of dissolved organic matter in AMD tends to be relatively low (< 20 mg l^−1^)^20^, but the total organic carbon of the AMD samples from the Onyeama coal mine was 25.7 ± 5.96 mg l^−1^ signifying oligotrophic conditions. Moreover, carbonate (253 ± 22.4 mg l^−1^) in the AMD indicated alkali stemming of acidic pH from the characteristic < 2 associated with freshly formed AMD to > 3.0 as presently observed.

A comprehensive assessment of HMs is pivotal to evaluating the potential of AMD to mitigate the degree of pollution in receiving environments. The contents of toxic metals and metalloid measured from the AMD water sample were extremely high, ranging from the Cr content (3.87 ± 3.87 mg l^−1^) to that of Pb (326.0 ± 26.8 mg l^−1^) (Supplementary Table A.1). Concentrations of Pb in AMD recorded in this study was higher than 12 mg l^−1^ associated with Iron Mountains’ AMD in the United States^21^ and 30 mg l^−1^ in Sao Domingo mine’s AMD in Portugal^23^. Other metals contained in the AMD include Cd (95.0 ± 5.12 mg l^−1^), Co (27.3 ± 9.25 mg l^−1^), Ni (28.8 ± 13.4 mg l^−1^), As (56.7 ± 14.7 mg l^−1^), and Fe (39.7 ± 22.3 mg l^−1^). All the metals/metalloids were apparently at toxic concentrations when compared with the WHO permissible limit and the values obtained from the unpolluted surface water located several kilometres away from the mine. Pb and Cd that connoted the highest concentrated HMs in the AMD have no metabolic importance other than rendering havoc to biota^8,24^ in ecosystems the AMD emptied. These non-metabolic HMs exacerbate ecophysiologies of the receiving milieus with anticipated degrees of public health. Such health consequences include mutagenicity, genotoxicity, neurotoxicity etc^5^. This was reportedly the case with surface waters juxtaposing with AMD from the Onyeama coal mine reportedly enriched with TOC and toxic concentrations of HMs^25^. Similarly, AMDs have reportedly remained one of the major point sources for anthropogenic HMs pollution of waters globally^2,11,12^.

The awful impact of metals/metalloid poised AMD on the receiving water quality is better modelled via integrating multivariate data into pollution indexes and the functionalities of the ecosystems (Table 1). The added HMs in the AMD from coal mine as determined by contamination factor (CF) was at least 397 (± 223) factors for Fe and up to 2.97 (± 0.16) × 10^6^ factors for Cd (Supplementary Table A.2). It implies an inordinate tendency of the AMD to contaminate water bodies. The added HMs was in the order: Cd > Co > Pb > As > Ni > Cr > Fe (Table 1). Enrichment of five HMs was exceptionally high (Cd > Co > Pb > As > Ni), while Cr and Fe were very high and moderately enriched the AMD water, respectively. The astronomically high contamination and enrichment factors of the AMD signified the enrichment potentials the AMD portends on receiving surface waters. The AMD from the Onyeama coal mine has been reportedly impacting the water qualities of rivers within the location^25^. It is assumed that the extremely high concentrations of toxic metals/metalloids in the AMD dilutes out upon discharges into nearby rivers, contaminating the surface water and raising the bioavailable metals/metalloids beyond safe thresholds. Further reports of toxic metals/metalloids enrichment of surface waters via inflow of AMDs from other mines in Nigeria^26^ and other climes^3,4,27^ are worrisome and oblige mitigations.

**Table 1:** Physico-chemistry, pollution and ecological impact determinants of heavy metals and metalloid contained in the AMD from coal mine.

Physico-chemistry	Measured values	WHO (1993) permissible limit
Temperature (°C)	27.2 ± 0.376	–
Colour (TCU)	393 ± 32.1	15
pH	3.1 ± 0.265	6.5–8.5
EC (µS cm^−1^)	139 ± 16.9	1400
Total dissolved solids (mg l^−1^)	132 ± 33.7	1000
Turbidity (NTU)	68.6 ± 21.0	5
Chlorides (mg l^−1^)	110 ± 57.5	250
Carbonate (mg l^−1^)	253 ± 22.4	–
SO_4_^2−^ (mg l^−1^)	313 ± 15.9	250
Sulphides (mg l^−1^)	1.37 ± 0.233	–
Total organic carbon (mg l^−1^)	25.7 ± 5.96	–
NO_3_^−^ (mg l^−1^)	86.6 ± 41.0	50
PO_4_^2−^ (mg l^−1^)	0.05 ± 0.021	5

Geochemical indexes	Determinants	Interpretations
**Pollution factors**		
CF	Very high (> 6)	Cd > Co > Pb > As > Ni > Cr > Fe
EF	Exceptionally high (> 50)	Cd > Co > Pb > As > Ni
	Very high (25 ≤ EF < 50)	Cr
	Moderate (3 ≤ EF < 5)	Fe
I_geo_	Very severe (≥ 5)	Cd > Co > Pb > As > Ni > Cr > Fe
PLI (× 10^2^)	31.1 ± 9.42	Progressively deteriorating (> 100)
PI (× 10^5^)	21.3 ± 1.16	Severe (> 3)
MPI (× 10^3^)	20.8 ± 1.19	Severe (> 10)
C_d_ (× 10^5^)	34.0 ± 2.40	Severe
MC_d_ (× 10^3^)	33.4 ± 2.44	Severe
**Ecological risks**		
Er	Very high (Er > 320)	Cd > Co > Pb > As > Ni > Cr > Fe
MEr	Very high (MEr > 320)	Cd > Co > Pb > As > Ni
	Considerate (80 < MEr ≤ 160)	Cr
	Low (MEr < 40)	Fe
RQ	High (RQ > 1)	Pb > Cd > As > Ni > Co > Fe > Cr
RI (× 10^6^)	38.1 ± 2.18	Very high (RI > 320)
MRI (× 10^4^)	37.5 ± 2.24	Very high (MRI > 320)

Values are mean (± SEM) of triplicate sampling measurements.

The pollution indexes and ecological risk assessment factors include: contamination factor (CF), enrichment factor (EF), geo-accumulation index (I_geo_), pollution load index (PLI), pollution index (PI), modified pollution index (MPI), degree of contamination (C_d_), modified degree of contamination (MC_d_), potential ecological risk factor (Er), modified potential ecological risk factor (MEr), potential ecological risk index (RI), modified potential ecological risk index (MRI), risk quotient (RQ).

The HMs-enriched environments inadvertently exert ecotoxicity unto the drivers of the ecosystems. The level of HMs accumulation to the organic matter in the AMD, through geo-accumulation (I_geo_) index of Fe (7.60 ± 0.779) to Cd (20.9 ± 0.075) (Supplementary Table A.2), was very severe and in a similar order to CF. It possibly implies organic matter in the AMD harbours the mobile toxic metal/metalloid concentrations and make them available to the food web^28^. Thus, biomagnification of the toxic metals/metalloids along the trophic level becomes palpable and a challenge to the biota of any surface water receiving the AMD and to public health^21,28^. Ecological risk assessments define and categorise the pollution status of ecosystems with the HMs contained in the AMD. Based on the potential ecological risk factor (Er), Cd exerted an extremely high-risk index (36.3 ± 1.96 × 10^6^), and none of the metals/metalloids exercised less than 1000 risk index (Supplementary Table A.2). All the HMs/metalloid contained in the AMD posed very high ecological risks and could be categorised in the order of Cd > Co > Pb > As > Ni > Cr > Fe. The modified potential ecological risk factor (MEr), however, stipulated that five HMs posed a very high risk in the order: Cd > Co > Pb > As > Ni, whereas Cr and Fe were determined to be of considerate and low risks, respectively. The HMs exerted high risk to the AMD ecosystem as calculated by ecological risk quotient (RQ) in the order: Pb > Cd > As > Ni > Co > Fe > Cr. The ecological risk index of all the HMs as a whole was very high (375,000 ± 22,400) index as stipulated by the modified potential ecological risk index (Table 1). The prodigiously high ecological risks indexes of the HMs/metalloid in the AMD indicated grave danger the AMD would portend on the surface- and ground-waters.

### Microbial community structure of AMD from Onyeama coal mine

A total of 26,160 and 40,403 valid (filtered) sequence reads were obtained for bacteria and eukarya, respectively, after a quality check of biofilm-water amplicon sequence data. The valid sequences were clustered into 2036 and 1002 operational taxonomic units (OTUs) of bacteria and eukarya domains of life, respectively, as presented in Table 2. Microbial community structures are sensitive descriptors of ecological stressors pivotal to understanding ecosystem functions^29^. The number of clustered high quality, non-chimeric sequences as OTUs based on UCLUST and CD-HIT against the sequence reads was depicted as asymptotic rarefaction curves (Supplementary Fig. A.1). The curves revealed that higher numbers of OTUs were delineated from valid sequence reads of 16S rRNA genes, unlike the lesser number of OTUs obtained from valid sequence reads of ITS2 region located between 5.8S and 28S rRNA genes of eukaryotes. The OTU richness observed in the rarefaction curves established coverage of the majority of species and was further validated with the richness and diversity estimations presented in Table 2. Despite the higher number of valid sequence reads obtained from the amplified ITS2 (40,403) than that of 16S rRNA genes (26,160), the observed OTUs were more in 16S rRNA genes (2036) than those of ITS2 (1002). More than 99.8% and about 98.5% of the microbial community in AMD from the Onyeama coal mine represented eukarya and bacteria OTUs, respectively, based on estimated Good’s library coverage. The coverage degree of the MiSeq sequencing corroborated the rarefaction curves. Furthermore, the estimated OTU richness (based on higher values obtained from ACE, Chao1 and JackKnife indexes) showed that bacterial phylotypes were richer than those of eukarya. Alpha diversity indexes (NPShannon, Shannon, and inverse Simpson) phylogenetic diversity index revealed that bacteria in the AMD were more diverse than eukarya OTUs.

**Table 2:** Alpha diversity of microbiome evenness, richness and varieties of species in the sediments.

	Bacteria		Eukarya
	Biofilm water (AMD-EB)	Enrichment culture (AMD-EC)	Biofilm water (AMD-EB)
**Actual**			
Valid reads	26,160	26,373	40,403
OTUs	2036	95	1002
**Estimated richness**			
ACE	2293.26	133.37	1033.01
HCI	2346.43	169.09	1047.66
LCI	2244.59	109.57	1019.11
Chao1	2174.97	126.23	1016.11
HCI	2217.70	173.29	1029.93
LCI	2142.28	107.46	1009.13
JackKnife	2438	127.45	1078
HCI	2438	145.15	1078
LCI	2438	109.75	1078
**Estimated diversity**			
NPShannon	5.97	1.58	3.54
Shannon	5.86	1.57	3.51
HCI	5.88	1.59	3.54
LCI	5.84	1.55	3.48
Simpson	0.01	0.41	0.13
HCI	0.01	0.42	0.14
LCI	0.0097	0.41	0.13
Phylogenetic diversity	2779	182	1186
Good’s Lib. coverage (%)	98.45	99.89	99.81

Clustering of OTUs found was achieved with UCLUST and the open reference method as all taxa were selected for analysis.

*HCI* High Confidence Interval (95%), *LCI* Low Confidence Interval (95%), *OTUs* Operational taxonomic units determined at 97%.

### Taxonomy and phylogeny of microbial OTUs in AMD from coal mine

**Figure 1:**
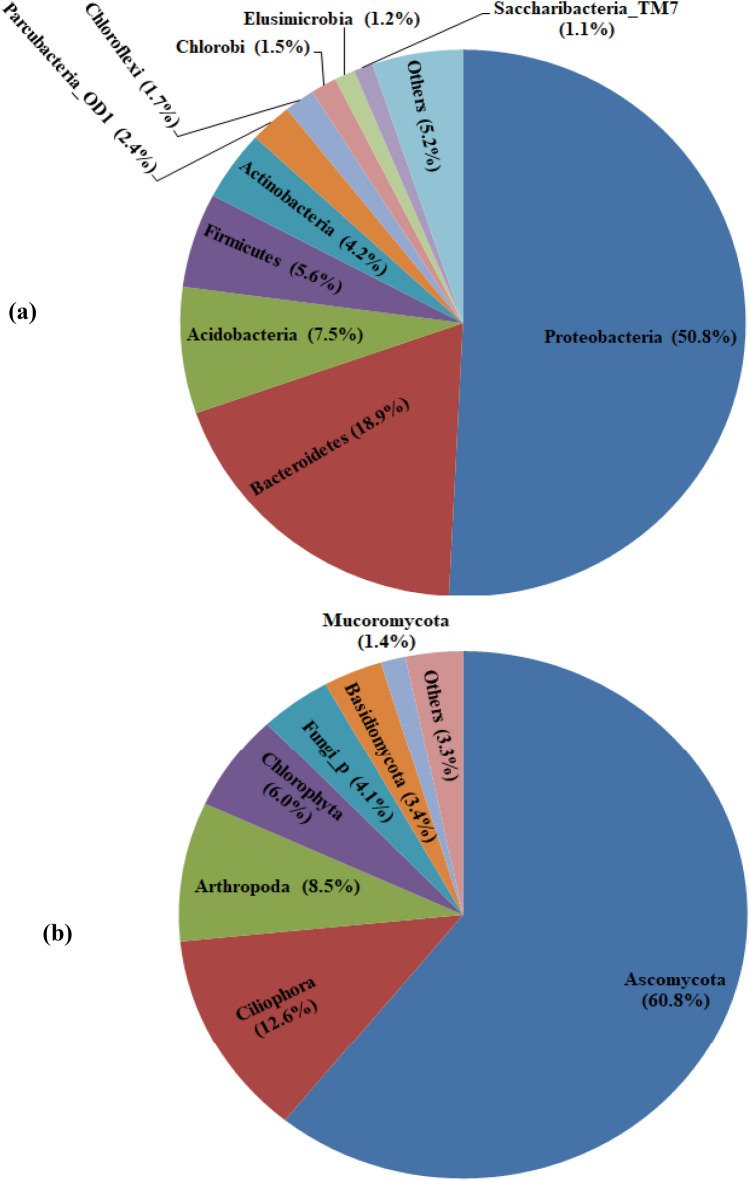
Taxonomic composition of Bacteria (**a**) and Eukarya (**b**) domains of life found in the AMD from Onyeama coal mine, showing specific phyla and sub-kingdoms for kingdoms Plantae and Animalia. Phyla and sub-kingdoms that are less than 1% of the total sequence reads were regarded as ‘Others’.

The taxonomic composition and relative abundances of the AMD microbiome, as shown in Fig. 1, revealed that the bacterial community spanned 10 phyla whose sequence reads were at least 1% (Fig. 1a). Whereas the eukarya domain of life (with sequence reads ≥ 1%) found in the AMD include Fungi, Plantae and Animalia kingdoms (Fig. 1b). Ascomycota, unclassified Fungi phylum (Fungi_p), Basidiomycota, and Mucoromycota represented Fungi kingdom, while Ciliophora and Arthropoda phyla were Animalia and Chlorophyta phylum epitomised Plantae kingdom. Association of the domain Eukarya (comprising Alveolates, Chlorophyta and Fungi as observed in this study) with AMD is reported to a lesser extent when compared with Bacteria^30^. The Fungi, largely represented by Ascomycota and Basidiomycota, are primarily found in sub-surface low-pH biofilms thriving in AMD^31^. While the Alveolates are suggested to have acted as primary/secondary consumers, the amoebae were secondary grazers in the AMD ecosystem^29,32^. Fungi taxa must have participated in carbon cycling as the main decomposers in the microbial community of the AMD. The taxonomic composition and relative abundance of phyla regarded as ‘Others’ (sequence reads < 1%) were presented in Supplementary Table A.3.

Among the phyla dominating the Bacteria domain of life in the Onyeama AMD were highly diverse classes that are known with AMDs. Low abundance Firmicutes and Actinobacteria lineages have also been previously characterized in AMD amplicon sequence data sets^6,10^). Previous studies revealed that oligotrophic *Leptospirillum* and archaea dominated the early stage of biofilm development in AMD^33^. Acidophilic copiotrophic heterotrophs comprising surprisingly wide diversity (physiology and phylogeny) with prevailing metabolic traits are known to succeed the early colonisers^34^. Interestingly, families in the Class Bacteroidia, including Porphyromonadaceae (12.4%), Prolixibacteraceae (1.6%), and unclassified GU454901 (1.2%), were dominant in the coal AMD as previously reported^10^. In Eukarya, dominant classes in the kingdom Fungi spread among Ascomycota (Eurotiomycetes, 33.8%; unclassified Ascomycota class, 18.1%; Dothideomycetes, 3.7%; Sordariomycetes, 3.3%; and Saccharomycetes, 1.3%), unclassified Fungi (4.0%), Mucoromycota (Umbelopsidomycetes, 1.2%), and Basidiomycota (Tremellomycetes, 1.2%, and Agaricomycetes, 1.0%). Dominant Animalia in the AMD comprised sub-kingdom Alveolata and Metazoa represented by unclassified Ciliophora (12.6%) and unclassified Arthropoda_c (8.4%), respectively. Metabolic CO_2_ from protozoan respiration is assumed to further increase the level of dissolved inorganic carbon contributing to carbonate concentration that curtailed acidic pH in the microenvironment^32^. Unclassified Chlorophyta class (5.5%) was the only taxonomic class of Phylum Chlorophyta belonging to kingdom Plantae that formed part of dominant Eukarya in the AMD.

**Figure 2:**
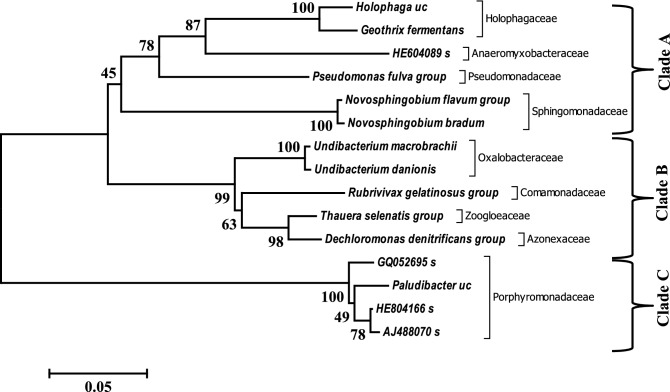
Evolutionary relationships of dominant bacteria taxa in the AMD from Onyeama coal mine. The evolutionary history was inferred using the Neighbor-Joining method. The percentage of replicate trees in which the associated taxa clustered together in the bootstrap test (1000 replicates) is shown next to the branches. The tree was drawn to scale, with branch lengths in the same units as those of the evolutionary distances used to infer the unrooted phylogenetic tree. The evolutionary history was inferred using the Neighbor-Joining method. Evolutionary analyses were conducted in MEGA6, and clades were determined based on monophyletic and polyphyletic grouping.

**Figure 3:**
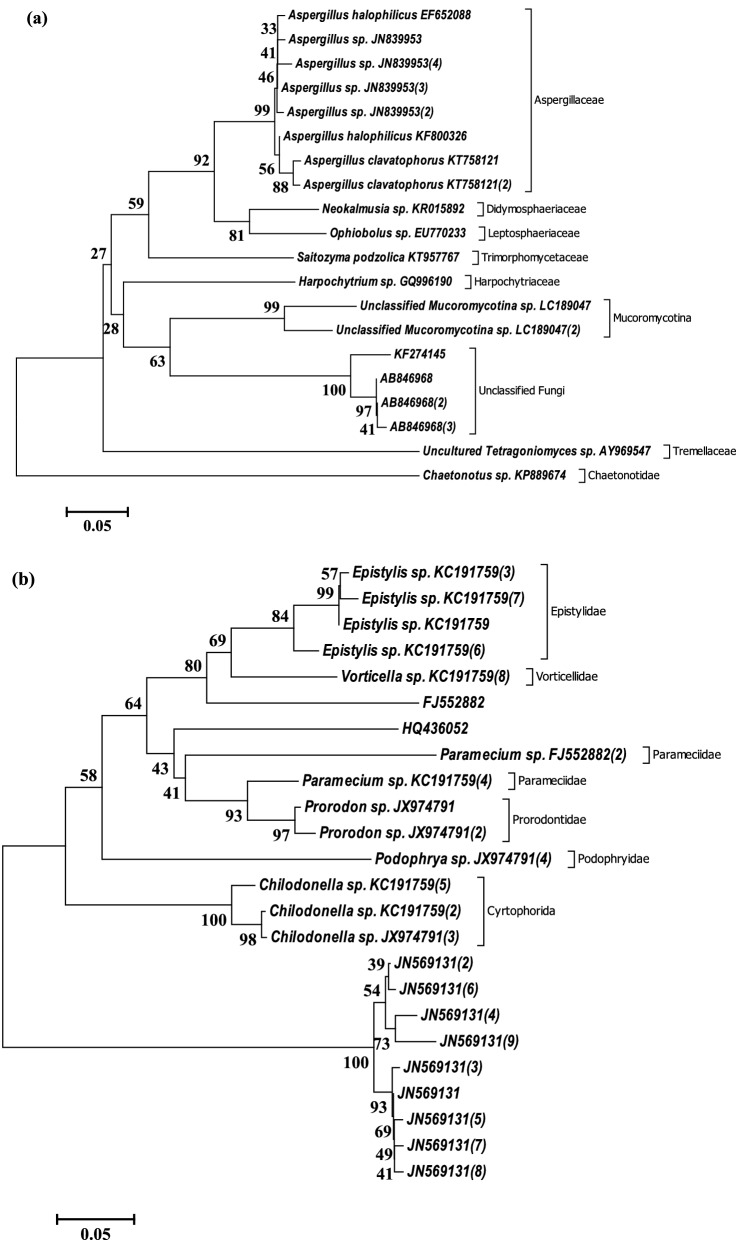

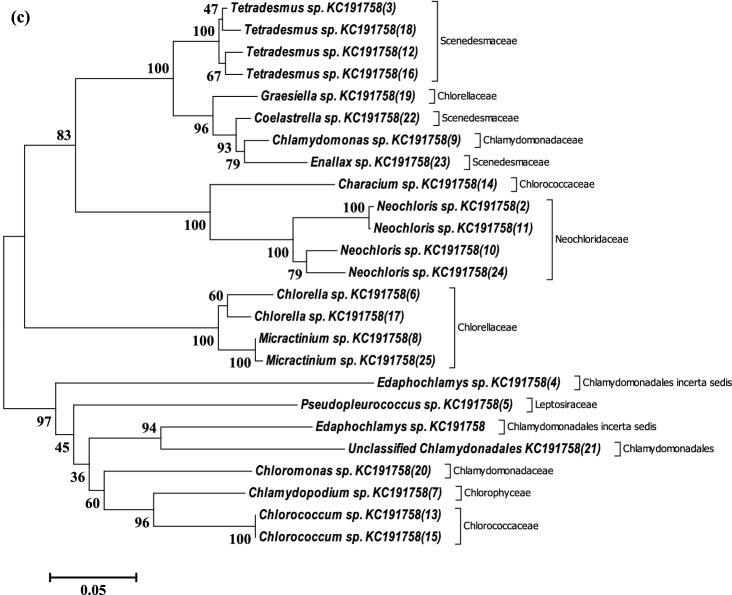
Evolutionary relationships of dominant OTUs of Eukarya showing selected strains of dominant Fungi (**a**), Animalia (**b**) and Plantae (**c**) taxa in AMD biofilm-water from ‘Onyeama’ coal mine. The evolutionary history was inferred using the Neighbor-Joining method. The percentage of replicate trees in which the associated taxa clustered together in the bootstrap test (1000 replicates) is shown next to the branches. The trees were drawn to scale, with branch lengths in the same units as those of the evolutionary distances used to infer the unrooted phylogenetic tree. The evolutionary distances were computed using the Maximum Composite Likelihood method Evolutionary analyses were conducted in MEGA6.

The phylogeny, based on evolutionary history, of bacterial OTUs, was deduced via the Neighbor-Joining method as an unrooted phylogenetic tree (Fig. 2). The bacterial OTUs have a relative abundance ≥ 1% of the total valid sequence reads. The dominant bacterial OTUs aligned into three clades was based on monophyletic and polyphyletic grouping. The OTUs have not been reported as dominating bacteria communities in AMD biofilm development other than transforming AMD^35^. The relative abundance of the bacterial OTUs is presented in Supplementary Table A.4. Species of *Paludibacter* are acidophilic and have been associated with the reduction of sulphate and Fe^3+^ in an AMD-impacted site^35^. Furthermore, *the Rubrivivax gelatinosus* group use NiFe hydrogenase to stem H^+^ to hydrogen. Whereas, *Novosphingobium flavum* group degrade coal hydrocarbons and generate hydrogen using formate dehydrogenase enzyme^36^. Moreover, *Thauera selenatis* is known for using selenate or other metals as the preferred electron acceptor for respiration. *Dechloromonas* species are famous for their denitrifying role in the extreme ecosystem^37^. Nevertheless, the dominant eukaryotic OTUs (sequence reads ≥ 0.5%) spread across Fungi (7 OTUs), Animalia (3 OTUs) and Plantae (3 OTUs) as presented along with their corresponding counts and ratio (see Supplementary Table A.5). The evolutionary relatedness of representative sequences (based on the abundance) of the OTUs was calculated and delineated as unrooted phylogenetic trees (Fig. 3). It is important to note that majority of the dominant OTUs delineated as Eukarya in the AMD were unclassified, and their role in carbon fluxes cannot be ascertained for now.

### Sequestration of toxic HMs/metalloid from simulated AMD and actual AMD from coal mine

**Figure 4:**
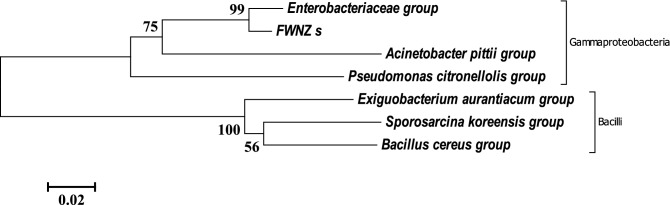
Evolutionary relationships of bacteria taxa that form consortium used in HMs sequestration of AMD from ‘Onyeama’ coal mine. The evolutionary history was inferred using the Neighbor-Joining method upon alignment via MUSCLE. The tree was drawn to scale, with branch lengths in the same units as those of the evolutionary distances used to infer the unrooted phylogenetic tree. The evolutionary distances were computed using the Maximum Composite Likelihood method with pairwise deletion and 1000 bootstrap replicates (bootstrap value > 50%). Evolutionary analyses were conducted in MEGA6.

Fortification of rich or semi-rich culture broth with toxicants of interest is a common approach to selecting competent microorganisms^38^. After culture enrichment, 26,373 valid reads from non-chimeric sequences were clustered into 95 OTUs of bacteria. Alpha diversity of the enriched culture revealed low species richness based on estimated values via ACE, Chao1 and JackKnife (Table 2). Estimated diversity further depicted poorly diverse bacteria species in enrichment culture via phylogenetic diversity valued at just 182. This also corroborated the estimates of other diversity indexes (NPShannon, Shannon, and inverse Simpson) presented in Table 2. Culture enrichment was biased towards a few OTUs that the culture conditions supported. On the contrary, a richer and more diverse community was observed in the coal AMD as depicted in the alpha diversity indexes. After clustering the valid sequence reads of 16S rRNA gene sequencing from clone library into OTUs, the consortium of bacteria containing 7 dominant groups of OTUs was observed to be involved in toxic metal sequestration of AMD. The taxonomy and counts at inoculation and post-incubation comprised two taxonomic classes of bacteria (Supplementary Table A.6), whose evolutionary relatedness was depicted as an unrooted phylogenetic tree (Fig. 4). The classes with their OTUs include ϒ-Proteobacteria (*Acinetobacter pittii* group, Enterobacteriaceae group, unclassified FWNZ species, and *Pseudomonas citronellolis* group), and Bacilli (*Sporosarcina koreensis* group, *Bacillus cereus* group, and *Exiguobacterium aurantiacum* group). The bacteria (particularly *Acinetobacter pittii, Pseudomonas citronellolis,* and *Bacillus cereus*) have been involved in the degradation of indole, and a heterocyclic aromatic compound found in coal, via an attack on either/or both the carbocyclic and *N*-heterocyclic rings^39^. Studies involving *Acinetobacter*^40^, Enterobacteriaceae^17^, *Pseudomonas*^41^, *Sporosarcina*^7,42^, *Bacillus*^42^, and *Exiguobacterium*^40^ OTUs for sequestration of toxic metals/metalloids in environmental media have been reported. Bioaccumulation of Cd, Co, and Zn was reported for *Sporosarcina* sp. G3 as sequestration strategy, whereas Cr and Hg were reduced via redox-active enzymatic activities to innocuous forms^43^.

**Figure 5:**
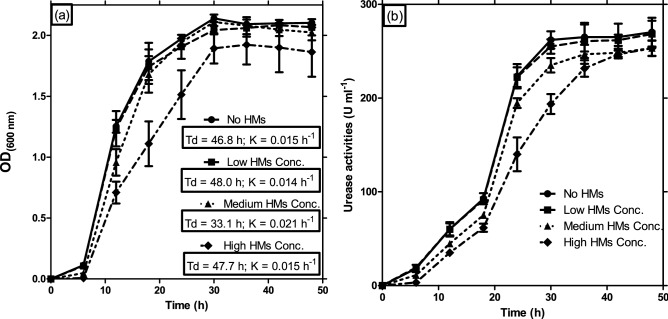
Growth kinetics of bacterial consortium via viable counts extrapolated into optical density at 600 nm wavelength (**a**) and growth-dependent urease activity of bacterial consortium (**b**) in TGYM broth without heavy metals (HMs) cocktail, and with low, medium, and high concentrations of HMs cocktails. Low HMs concentrations cocktail comprised (per liter) Cd, 27.9 mg; Pb, 118.7 mg; Co, 16.2 mg; Ni, 16.2 mg; and As, 61.5 mg. While medium HMs concentration contained (per liter) Cd, 55.7 mg; Pb, 237.3 mg; Co, 32.4 mg; Ni, 32.3 mg; and As, 123.1 mg. High HMs concentration contained (per liter) Cd, 139.3 mg; Pb, 593.3 mg; Co, 81.1 mg; Ni, 80.7 mg; and As, 307.6 mg. The mean pH at the beginning of experiment was 3.5 and rose to 8.2–8.4 at 48 h post-inoculation. Growth kinetics at exponential growth phase are in the inserts of panel (**a**), where ‘Td’ represents doubling time and ‘K’ is the growth rate at exponential growth phase. Error bars represent standard error mean (SEM) of triplicate experiments. The culture conditions were as explained in the “Methods” Section (Growth kinetics and urease activity of bacteria consortium; Determination of bacterial growth-dependent HMs/metalloid sequestration in simulated and natural AMD).

Urease-producing bacteria instigate insoluble metal-carbonate micro-precipitation through urease activity^16^. The growth-time courses and urease activities of the bacteria consortium in simulated AMD were presented as curves (Fig. 5). It was observed that the impact of high concentrations of HMs cocktails was not pronounced beyond the early 6 h post-inoculation, which was regarded as the lag phase. The bacteria consortium might have activated necessary genes needed to tolerate and sequester the metals/metalloids toxicity during the lag phase without cell multiplications. Afterwards, the bacteria consortium grew steadily with the production of urease, based on increasing measurement of urease activity, as incubation continued. At 30 h post-inoculation, 245.3 (± 23.7) U ml^−1^ activity of urease was observed in broth without a toxic metal cocktail. However, more urease activity (255 ± 7.6 U ml^−1^) by the bacteria consortium was observed in medium amended with low concentrations of metal cocktails unlike lesser activities of 235 (± 7.6) U ml^−1^ and 193.7 (± 10.7) U ml^−1^ associated with medium and high metal concentrations, respectively. As the growth remains stationary and pH further increased to > 8.2, urease activities were at least 253 U ml^−1^ in all the cultures. Although urease activities at acidic pH have been reported in acid-tolerant human pathogens^19^, the findings in this report were assumedly the first amongst bacterial strains from AMD-polluted environments. The urease activities at acidic pH compared favourably with activities at alkaline pH in previous studies^7,16,42,44^. Moreover, the pH of the culture system kept increasing, alleviating the acidity condition that initially prevailed in the AMD system.

Interestingly, urease activity was observed in low quantity at acidic pH, unlike higher activity when the pH inclined towards alkaline (Fig. 5). It is proposed that urea finds its way into Onyeama coal mine drains through runoff from agricultural soils fortified with urea fertilizers and animal manures, which are common agricultural practices in Nigeria. The products of urea hydrolysis might have equilibrated in water to form bicarbonate, ammonium and hydroxyl ions that serially increased the culture pH. Ultimately, the bicarbonate equilibrium might have shifted to form carbonate ions (HCO_3_^−^ + H^+^ + 2NH_4_^+^ + 2OH^−^ ↔ CO_3_^2−^ + NH_4_^+^  + 2H_2_O) that enhanced the metal-carbonate micro-precipitation (Me^2+^  + Cell → Cell-Me^2+^ + CO_3_^2−^ → Cell-MeCO_3_). The gradual increase in pH could have further indulged the formation of CO_3_^2−^ from HCO_3_^−^, leading to metal-CO_3_ precipitation around cells and in culture media. Bicarbonates enrichment with inherent ammonia production was thought to have provided additional acid neutralization of the AMD. The growth kinetics after the presumed lag phase in the early 6 h to late exponential phase at 18 h showed that a low concentration of HMs cocktails did not have an impact on the growth of the bacteria consortium. Consequently, the bacteria consortium exhibited excellent sequestration of multi-component toxic HMs in both the simulated toxic metal-rich AMD and the actual AMD obtained from the Onyeama coal mine (Table 3).

**Table 3:** Growth associated sequestration and precipitation of heavy metals/metalloid cocktail and AMD from Onyeama coal mine.

	Sequestration efficiency at 24 h incubation (%)					Precipitate weight (mg ml^−1^)
	Cd	Pb	Co	Ni	As	ΣHMs
Low HMs^a^	100	100	100	95.9 ± 4.1	91.3 ± 3.3	6.23 ± 0.43
Medium HMs^b^	100	100	84.6 ± 6.4	89.7 ± 10.3	87.0 ± 2.6	11.9 ± 0.97
High HMs^c^	100	100	76.6 ± 3.8	89.7 ± 10.3	84.8 ± 2.3	15.6 ± 0.92
Natural AMD^d^ + Urea	100	100	70.1 ± 26.8	95.2 ± 4.8	91.2 ± 1.69	10.5 ± 0.52
Natural AMD only	96.3 ± 3.18	94.8 ± 2.46	76.9 ± 2.99	90.6 ± 2.76	88.9 ± 0.982	8.57 ± 2.52

Values are mean (± SEM) of triplicate experiments.

^a^Low HMs broth comprised (per liter) Cd, 27.9 mg; Pb, 118.7 mg; Co, 16.2 mg; Ni, 16.2 mg; and As, 61.5 mg.

^b^Medium HMs broth contained (per liter) Cd, 55.7 mg; Pb, 237.3 mg; Co, 32.4 mg; Ni, 32.3 mg; and As, 123.1 mg.

^c^High HMs broth contained (per liter) Cd, 139.3 mg; Pb, 593.3 mg; Co, 81.1 mg; Ni, 80.7 mg; and As, 307.6 mg.

^d^At the start of experiment, Natural AMD from coal mine used in this study contained (per liter) Cd, 95.0 ± 5.12 mg; Pb, 326 ± 26.8 mg; Co, 27.3 ± 9.25 mg; Ni, 28.8 ± 13.4 mg; and As, 56.7 ± 14.7 mg.

The bacteria consortium displayed more than 94% efficiency of Cd and Pb sequestration in natural AMD, while 100% efficiency was observed in all the simulated AMD treatments (Table 3). Low performance was found with Ni and As, but not less than 70% sequestration efficiency was observed in all treatments. Efficient sequestrations of toxic metals, up to 100% removal efficiency of most toxic metals, observed with the bacteria consortium were similar to findings in a previous study^13^. Mixed-bacterial cultures are known to be able to perform more complex tasks and survive in more unstable environments than a monoculture. Nevertheless, 89.3–98% removal efficiencies of Ni, Pb, Co, and Cd from solution have been reportedly achievable with urease-producing *Sporosarcina koreensis*^45^. Similarly, *Bacillus* sp. KK1 reportedly mitigated lead-contaminated mines tailings containing mobile Pb (1050 mg kg^−1^) to form insoluble precipitates of PbS and PbSiO_3_^34^. Growth-dependent sequestration of HMs cocktails by the bacteria consortium was adduced to be via precipitation. The weight of the precipitates was evaluated to be proportional to concentrations of HMs cocktail present. The bacteria consortium was observed to drive the formation of as much as 15.6 (± 0.92) mg ml^−1^ precipitates (Table 3) that were assumed to be in form of HMs-carbonates in TGYM supplemented with high concentrations of HMs cocktail within 24 h post-inoculation. In natural AMD bio-stimulated with urea and seeded with bacteria consortium for 24 h, 10.5 (± 0.52) mg ml^−1^ HMs precipitates was observed unlike 8.57 (± 2.52) mg ml^−1^ precipitates obtained from natural AMD toxic metals sequestration without urea fortification. It appeared that the quantity of toxic metal precipitate was proportional to quantities of available toxic metals, which corresponded to the number of heterogeneous nucleation sites on the surface of the bacterial cells. Omoregie et al.^42^ reported a relatively similar quantum of precipitation as CaCO_3_ with species of ureolytic Firmicutes isolated from limestone caves. As such, there was no correlation between urease activity and quantum of toxic metal precipitation since there is a likelihood that other metabolic activities may be linked to urease activities. Nevertheless, the bioremediation strategies demonstrated in the present study exhibited excellent toxic metal sequestrations unlike insignificant (p > 0.05) natural attenuation process of the autochthonous community without augmentation with bacteria consortium and stimulation with nutrients (as presented in Table 3).

In conclusion, AMD from the Onyeama coal mine is a point source of pollution to the surrounding environments because of its richness in anions and toxic metals/metalloids. It has a high potential of enriching the receiving hydrosphere with toxic metals/metalloids and exerts severe ecological risks (Er > 320) with Cd and Pb wielding a huge critical risk index (38.1 ± 2.18 × 106) on the biological elements of the ecosystems. The dominance of Proteobacteria (50.8%), Bacteroidetes (18.9%), Ascomycota (60.8%), and Ciliophora (12.6%) characterised the microbial community of the AMD, where unclassified OTUs occurred mostly among the species. Enrichment of the AMDs skewed the bacterial community as depicted in the alpha diversity indexes against that of coal AMD leading to the selection of bacteria consortium with an excellent potential of stemming the toxicants in the AMD. The bacteria consortium efficiently removed toxic metals/metalloids (> 70%) through precipitation and simultaneously neutralised AMD acidity. The bacteria consortium exhibited appreciable urease activity (> 190 U ml^−1^), through which the precipitation was assumed possible via the formation of metal/metalloid-carbonates. The bacteria consortium is suggested to be a sustainable biotechnological candidate in designing a bioremediation strategy for decommissioning AMD before discharge into the surrounding environment.

## Methods

### Study site and sampling

Nigeria has more than 1200 million tons of coal reserves, most deposits in Enugu State, and had previously depended on coal for energy until the discovery and boom of petroleum. Onyeama coal mine is one of the derelict coal fields in Enugu State that has been inactive for more than five decades. Onyeama coal mine drainage was the location for the present study (GPS coordinates: Lat., 6° 26′ 28.1649″ E and Long., 7° 28′ 45.156″ E). Biofilm samples of AMD were randomly collected from drains emanating from the Onyeama coal mine. Biofilms on cobbles were removed using a soft bristle brush into the water in a sterile beaker. Biofilms that grow at the solution-air interface in AMD effluent were collected from the surface of AMD solutions in sterilised sampling bottles. A freshwater stream that has no history of contamination in the same locality was collected in sterile glassware. The freshwater served as reference background geochemical values used in calculating pollution and ecological risk indexes of the AMD. The samples (from 10 random points, approx. 10 m apart within a 100 m radius of the sampling location) were mixed to represent each sample of the three replicate sampling days (Jan. 17; Feb. 18, and Mar. 18, 2019). The samples were packed in Ziploc bags and transported to the laboratory in an ice cooler. Samples for microbiological culturing were stored at 4 °C, while those for geochemical analysis were treated with 17% EDTA and vortexed to disrupt biofilm in the AMD to form AMD-biofilm solutions based on modified methods of De Almeida et al.^46^. The AMD-biofilm solution for microbiome was pelleted (7000×*g*; 10 min), and all samples were stored at 4 °C (for microbial culturing) and − 40 °C (for geochemical analyses) until when needed.

### Geochemistry of AMD from coal mine and evaluation of hazardous metal pollution

Physico-geochemical parameters of the samples were determined using standard methods earlier reported^28,47^. The pH, colour and temperature were determined in situ using established protocols. Other assays including electrical conductivity, turbidity, chlorides, total dissolved solids (TDS), NO_3_^−^, SO_4_^2−^, PO_4_^2−^, CO_3_^−^, total organic carbon (TOC), and dissolved sulphides were determined ex situ using standard protocols. Seven HMs including Pb, Cd, Co, Ni, As, Fe, and Cr were quantified via Atomic Absorption Spectrophotometry (AAS) (AAS-Perkin-Elmer Analyst 200; Perkin-Elmer, Canada) after acidic digestion (HNO_3_/HClO_4_ [4:1, v/v]) of a sample (0.1 g) in a microwave oven^24^. Normalisation, validation, operational conditions, and the limit of detection of the AAS were as earlier reported^47^. The wavelengths used for Cd, Pb, Co, Ni, Cr, Fe and As measurements were 228.8, 283.3, 240.7, 231.1, 357.9, 248.3 and 193.7 nm analytical lines, respectively.

The pollution indices of the measured HMs involved determining contamination factor (CF), enrichment factor (EF), geo-accumulation (I_geo_), pollution load index (PLI), pollution index (PI), and degree of contamination (Cd) as earlier reported^28^. Moreover, the modified pollution index (MPI) and modified degree of contamination (MCd) were as stated (see Supplementary Calculations online for details). Potential ecological risk factor (Er) and potential ecological risk index (RI) determined the ecological risks of the measured HMs in the coal AMD as reported^28^. However, modified potential ecological risk factor (MEr), modified potential ecological risk index (MRI), and risk quotient (RQ) was calculated (see Supplementary Calculations online for details). All measurements and calculations were performed in three replicates.

### Microbiome analysis of AMD from coal mine

#### Isolation of total community DNA in AMD

Approximately 0.5 g dry weight of AMD-biofilm pellets was subjected to total community DNA (tcDNA) extraction, using FastDNA^®^ Spin Kit for Soil (MP Biomedicals, Solon, OH, USA) by following the manufacturer instructions, and mechanical lysis of cells was achieved with the FastPrep^®^ Cell Disruptor FP 120 (Qbiogene, Heidelberg, Germany). Interference of PCR with humic substances in tcDNA was prevented by adding 20 mg of sterile skim milk (Sigma-Aldrich) to sediment sample (0.5 g) in the lysing matrix as earlier reported^28^. The Cell Disruptor was operated at 6.5 speed for 40 s in order to achieve a harsh cell wall disruption. The yield, quality and fragment of the crude and purified tcDNA was checked with Nano-Drop Spectrophotometry along with 0.8% (w/v) agarose gel electrophoresis and visualized in UV light upon staining with ethidium bromide.

### High throughput sequencing and amplicon sequence data analysis

Using tcDNA as a template, the V3–V4 region of 16S rRNA genes (bacteria) and ITS (Internal Transcribed Spacer) genes between 5.8S and 28S rRNA genes (eukarya) were amplified using primer set 341F and 805R, and ITS3-Mi (forward) and ITS4-Mi (reverse), respectively (please see Supplementary Method A.1 in the supplementary document for sequences of primers and PCR conditions). Libraries were constructed (as illustrated in Supplementary Method A.1) where qualities of the library were checked with Agilent 2100 Bioanalyzer System (Agilent Technologies, Palo Alto, CA, USA) using a DNA 7500 chip and quantified using Quanti-iT™ PicoGreenTM dsDNA Assay kit (Invitrogen) according to the manufacturer’s instructions. Short DNA fragment was removed using CleanPCR™ (CleanNA, Netherlands), and sequencing was performed via Illumina MiSeq platform (Illumina, San Diego, CA, USA), using Illumina MiSeq Reagent Kit v2 (500-cycles) at ChunLab Inc.

The quality of sequencing data was checked, and low quality (< Q25) reads were filtered with Trimmomatic 0.32 software^48^. The pair-end sequence of the same strand of PCR amplicon was merged based on overlapping sequence information using PANDAseq software^49^. ChunLab’s pipeline in-house algorithms were used to remove 16S rRNA PCR primer sequences, and UNITE (https://unite.ut.ee) was used to analyse the ITS2 gene. Non-specific amplicons were identified and removed using the HMMER program-based search to exclude Singleton sequences^50^. Denoising of sequences was performed with DUDE-Seq software^52^. The denoised sequences were de-replicated, and non-redundant reads were extracted via UCLUST-clustering^50^. UCHIME was used for the detection and removal of chimaera against BIOiPLUG’s chimaera-free reference database. The remaining non-chimeric sequences were clustered into operational taxonomic units (OTUs) using UCLUST as discussed by Lee et al.^51^. Taxonomic assignment was by comparing the sequence reads against the EzBioCloud 16S database (https://www.ezbiocloud.net/), using a combination of the initial BLAST-based searches, and additional pairwise 97% similarity comparisons as the cut-off^52^. The estimated coverage of the constructed 16S rRNA gene libraries was calculated as: $C=1-\left(\frac{n}{N}\right)\times 100$ according to Kemp and Aller^53^, where n is the number of Singletons after assembly of overlapping non-chimeric sequence reads and N is the total number of sequences in the initial dataset. Richness and diversity statistics of the bacterial community including abundance-based coverage estimator (S_ACE_), the bias-corrected Chao1 (S_Chao1_) and the Shannon–Weaver diversity index were estimated using a pre-calculated program of CLcommunity™ software package (ChunLab Inc.). Non-chimeric amplicon sequences were delineated into rarefaction curves upon UCLUST clustering into fasta format sequence data followed by Cluster Database at High Identity with Tolerance (CD-HIT) program incorporated in the CLcommunity™ software.

### Culture enrichment and toxic metal sequestration using bacterial consortium

A consortium of bacteria indigenous to coal AMD that tolerate elevated concentrations of HMs mixtures were sought in an attempt to develop a bacteria-based bioremediation strategy for alleviating HM-toxicity in AMD. These involved.

### Mixed-culture conditions and identification of bacteria consortium tolerant to HMs

The AMD-biofilm sample (10 ml) was enriched in a modified sterile Tryptone Glucose Yeast extract (TGY) broth (90 ml) containing (l^−1^): casein peptone, 5 g; glucose, 1 g; yeast extract, 2.5 g (Xebios Diagnostics, Düsseldorf, Germany), dissolved in mineral salts solution instead of distilled water to form Tryptone Glucose Yeast extract (TGYM) broth. The mineral salts solution contained (l^−1^): K_2_HPO_4_, 1.775 g; KNO_3_, 2 g; NaCl, 2 g; MgSO_4_. 7H_2_O, 0.05 g; CaCO_3_, 0.02 g; FeSO_4_·7H_2_O, 0.01 g, and pH adjusted to 3.5 (approx.) concerning the highest measured pH of AMD from the Onyeama coal mine. TGYM broth was amended with 10 mg l^−1^ CdCl_2_, 20 mg l^−1^ PbCl_2_, 10 mg l^−1^ CoCl_2_, 10 mg l^−1^ NiCl_2_, and 20 mg l^−1^ Na_2_HAsO_4_. The glucose solution was filtered through 0.2 µm Minisart syringe filters (Sartorius Stedim Biotech, Gottingen, Germany) and added aseptically. Culture enrichment was achieved via four transfers and incubations (30 °C; 100×*g*; 48 h), after which 10 ml culture was harvested (10,000×*g*; 10 min) and washed with phosphate-buffered saline twice. The washed biomass of bacteria consortium was resuspended (approx. 10^9^ cells ml^−1^) in sterile buffered saline and stored in glycerol (1:1, v/v) mixture at − 20 °C. Total DNA (tDNA) was extracted from broth culture biomass, bacteria domain amplified based on V3–V4 region of 16S rRNA gene, library constructed and sequenced using Illumina MiSeq system and taxonomic assignments of non-chimaera sequences were as explained in high throughput sequencing and metagenome data analysis section.

### Preparation of bacteria consortium biomass for sequestration of toxic metals mixture

Before further studies, bacteria consortium biomass was resuscitated from the stock by pre-culturing in Erlenmeyer flask containing TGYM broth for 24 h at 30 °C and 100×*g*, and the biomass was harvested (7000×*g*; 10 min), washed thrice with phosphate buffer (50 mmol l^−1^ KH_2_PO_4_, pH 7.2), and suspended in the same buffer. Resuscitated bacteria consortium (approx. 10^6^ CFU ml^−1^) were starved overnight in Tris–HCl (0.1 mol l^−1^) and re-suspended in sterile Milli-Q water (previously supplemented with mixtures of HMs: 5 mg l^−1^ each of CdCl_2_, PbCl_2_, CoCl_2_, NiCl_2_, and Na_2_HAsO_4_, final concentration) as inoculum.

### Growth kinetics and urease activity of bacteria consortium

Growth kinetics of the bacteria consortium was performed in TGYM broth (100 ml) supplemented with HMs mixture (5 mg l^−1^ each of CdCl_2_, PbCl_2_, CoCl_2_, NiCl_2_, and Na_2_HAsO_4_, final concentration) with and without urea (0.1 M final concentration) fortification, and pH adjusted to 3.5 (approx.). Inoculum (1 ml) as determined in the preparation of bacteria consortium section above, was added to the set-ups and incubated (100×*g*; 30 °C; 48 h). OD_(600 nm)_ and pH were measured at every 6 h post-inoculation. The urease activity of the mixed culture was determined as reported previously^7^, using phenol-hypochlorite assay where NH_4_Cl (50–1000 µM) was used as standard. Readings at 6 h intervals were as described^44^, and a unit of urease activity (U) was defined as the amount of urease hydrolysing 1 µmol urea min^−1^.

### Determination of bacterial growth-dependent HMs/metalloid sequestration in simulated and natural AMD

For simulated AMD, two sets of 500 ml Erlenmeyer flasks containing 100 ml of TGYM broth amended with HMs/metalloids cocktails (none, low, mid, and high concentrations; see Supplementary Table A.7 for details) were prepared, divided into two sets where a set was without urea and the other was fortified with urea (2%, w/v). Inoculum of bacteria consortium (1 ml), as prepared above was added into broth and incubated (100×*g*; 30 °C; 48 h). Growth of bacteria consortium was monitored every 6 h as turbidity via OD_(600 nm)_ of 5.0 ml sample using UV–visible spectrophotometer, and the pH was measured simultaneously. The blank was un-inoculated experimental set ups. To determine HMs/metalloid sequestration, biomass of each culture was harvested (10,000×*g*, 10 min), and supernatant was analysed for HMs/metalloid contents using AAS (as explained in Geochemistry of AMD Section above). The sequestration efficiency (SE) was determined using: $\frac{\left(MQM-SQ\right)}{MQM}\times 100$, where MQM is metal concentration in medium before inoculation, SQ is metal concentration in the supernatant after incubation^38^. Evidence of HMs/metalloid sequestration was determined by estimating HMs/metalloid-precipitation using modified methods of estimating CaCO_3_ precipitates earlier reported^42^. As such, TGYM broth was supplemented with urea 2% (w/v) and salts of HMs/metalloids instead of CaCl_2_, inoculated and incubated accordingly. Cultures were centrifuged (10,000×*g*; 60 s) to obtain metal-carbonate (Me-CO_3_) precipitates, washed twice with sterile dH_2_O (pH, 8.5), and air-dried (37 °C; 24 h), and weighted (dry weight per culture volume) to represent estimated amount of Me-CO_3_ mineral precipitated. Experimental set-ups but without bacterial inoculation, and TGYM without HMs/metalloid but inoculated with bacteria consortium were used as negative and positive controls.

For HMs/metalloid sequestration of natural AMD, drains (AMD sample) collected from Onyeama coal mine was filtered first through cellulose filter paper (Whatman™ 1001-070 Grade 1; pore size, 11 µm) and then serially through sterile Minisart syringe filters (0.45 µm → 0.2 µm, sequentially). The sterile AMD, without adjusting its pH but aseptically enriched with sterile casein (5 g l^−1^) and yeast extract (0.5 g l^−1^), was divided into four parts of 100 ml AMD in 500 ml Erlenmeyer flasks. Two of the flasks containing sterile AMD were aseptically supplemented with separately autoclaved urea (2%, w/v), while the other two flasks of AMD were without urea treatment. A flask of AMD with urea fortification and another without urea amendment were inoculated with bacteria consortium as stated earlier. Control experiments were un-inoculated AMD with and without urea. Both experimental set-ups and controls were incubated (100×*g*; 30 °C; 48 h). HMs/metalloid sequestration efficiency of the AMD was determined and HMs/metalloid precipitates were measured as explained earlier. Heterogeneous nucleation sites on bacterial cell surfaces were quantified according to the methods of Omoregie et al.^42^. All experiments were performed in three replicates.

### Statistical analyses and metadata achieving

The mean of three replicates of experimental values or measurements and standard error of the mean (SEM) was performed using the Prism 5 software program (GraphPad Software, San Diego, CA, USA). All charts (except pie chart that was performed using Microsoft Excel package) and curves were performed using Prism 5 software unless otherwise stated. All statistical tests were considered significant at p < 0.05. The sequencing metadata obtained and used in this study have been deposited in the NCBI’s sequence read archive (SRA) database under BioProject ID: PRJNA625753 under the BioSample accession SAMN14608860 (https://www.ncbi.nlm.nih.gov/biosample/14608860).

## Supplementary Information

Supplementary Information.
