# Supplementary material for: Microbiome of highly polluted coal mine drainage from Onyeama, Nigeria, and its potential for sequestrating toxic heavy metals
Source: Sci Rep. 2021 Sep 1;11:17496. doi: 10.1038/s41598-021-96899-z (PMC8410811; doi:10.1038/s41598-021-96899-z)
Supplement: Supplementary file 1 — Supplementary Information. [file 41598_2021_96899_MOESM1_ESM.pdf]

Table A.1: Geochemical properties of AMD water from 'Onyeama' coal mine.

Heavy metals/metalloids	Measured values (mg l^{-1})	WHO (1993) permissible limit (mg l^{-1})	Average contents in unpolluted freshwater (μg l^{-1})
Cadmium (Cd)	95.0 ± 5.12	0.003	0.032
Lead (Pb)	326 ± 26.8	0.01	3.0
Cobalt (Co)	27.3 ± 9.25	0.02*	0.1
Chromium (Cr)	3.87 ± 1.63	0.05	1.0
Nickel (Ni)	28.8 ± 13.4	0.02	1.5
Arsenic (As)	56.7 ± 14.7	0.01	2.0
Iron (Fe)	39.7 ± 22.3	0.3	100

*Co is rarely found in natural waters; therefore the permissible limit for Ni was adopted for Co
Values are mean (± SEM) of triplicate measures.

Table A.2: The pollution indexes and ecological risk assessment factors of AMD from 'Onyeama' coal mine

Heavy metals	**Pollution indexes**			**Ecological risk indexes**		
	**CF (× 10^3)**	**EF (× 10^2)**	**I_{geo}**	**Er (× 10^3)**	**MEr (× 10^2)**	**RQ**
Cd	2970 ± 160	292 ±16.5	20.9 ± 0.075	36300 ± 1960	3580 ± 203	1230 ± 67.3
Pb	109 ± 8.93	10.6 ± 0.875	16.1 ± 0.117	543 ± 44.6	54.6 ± 7.23	1270 ± 104
Co	273 ± 92.5	26.8 ± 9.07	17.3 ± 0.46	863 ± 292	84.6 ± 28.7	53.1 ± 18.0
Cr	3.87 ± 1.63	0.379 ± 0.159	10.9 ± 0.864	12.0 ± 5.12	1.20 ± 0.504	3.01 ± 1.27
Ni	19.2 ± 8.91	1.88 ± 0.874	13.4 ±0.638	60.7 ± 28.1	5.96 ± 2.76	56.1 ± 26.0
As	28.3 ± 7.33	2.78 ± 0.719	14.1 ± 0.344	283 ± 73.3	27.8 ± 7.19	220 ± 57.0
Fe	0.397 ± 0.223	0.039 ± 0.022	7.60 ± 0.779	1.25 ± 0.704	0.123 ± 0.069	5.15 ± 2.89

Values are mean (± SEM) of triplicate sampling measurements.
The pollution indexes and ecological risk assessment factors include: contamination factor (CF), enrichment factor (EF), geo-accumulation index (I_{geo}), potential ecological risk factor (Er), modified potential ecological risk factor (MEr), and risk quotient (RQ)

Table A.3: Taxonomic profile of sequence reads whose relative abundance were less than 1% in AMD from Onyeama coal mine

Bacteria		Eukarya	
Phylum	Ratio (%)	Phylum	Ratio (%)
Spirochaetes	0.948	Monoblepharomycota	0.916
Verrucomicrobia	0.933	Chytridiomycota	0.668
Gemmatimonadetes	0.787	Rozellomycota	0.418
Nitrospirae	0.482	Glomeromycota	0.384
Deinococcus-Thermus	0.386	Cercozoa	0.220
Planctomycetes	0.386	Blastocladiomycota	0.208
Cyanobacteria	0.168	Apicomplexa	0.124
Fusobacteria	0.138	Chromista_p	0.116
FJ437850_p	0.138	Mortierellomycota	0.072
Armatimonadetes	0.115	Plantae_p	0.047
WS5	0.111	Kickxellomycota	0.032
Omnitrophica_OP3	0.103	Fungi_uc	0.025
JMYB36	0.073	Eukarya_uc_p	0.017
Latescibacteria_WS3	0.065	Bacillariophyta	0.010
Peregrinibacteria	0.061	Metazoa_p	0.007
Chlamydiae	0.042		
SR1	0.038		
Kazan	0.038		
Microgenomates_OP11	0.031		
AY345499_p	0.027		
Caldithrix_p	0.023		
TDNP	0.015		
TM6	0.015		
BRC1	0.011		
Tenericutes	0.008		
Cloacamonas_p	0.008		
Aminicenantes_OP8	0.008		
EU266861_p	0.008		
Fibrobacteres	0.008		
GU390861_p	0.008		
HM128617_p	0.004		
GN04	0.004		

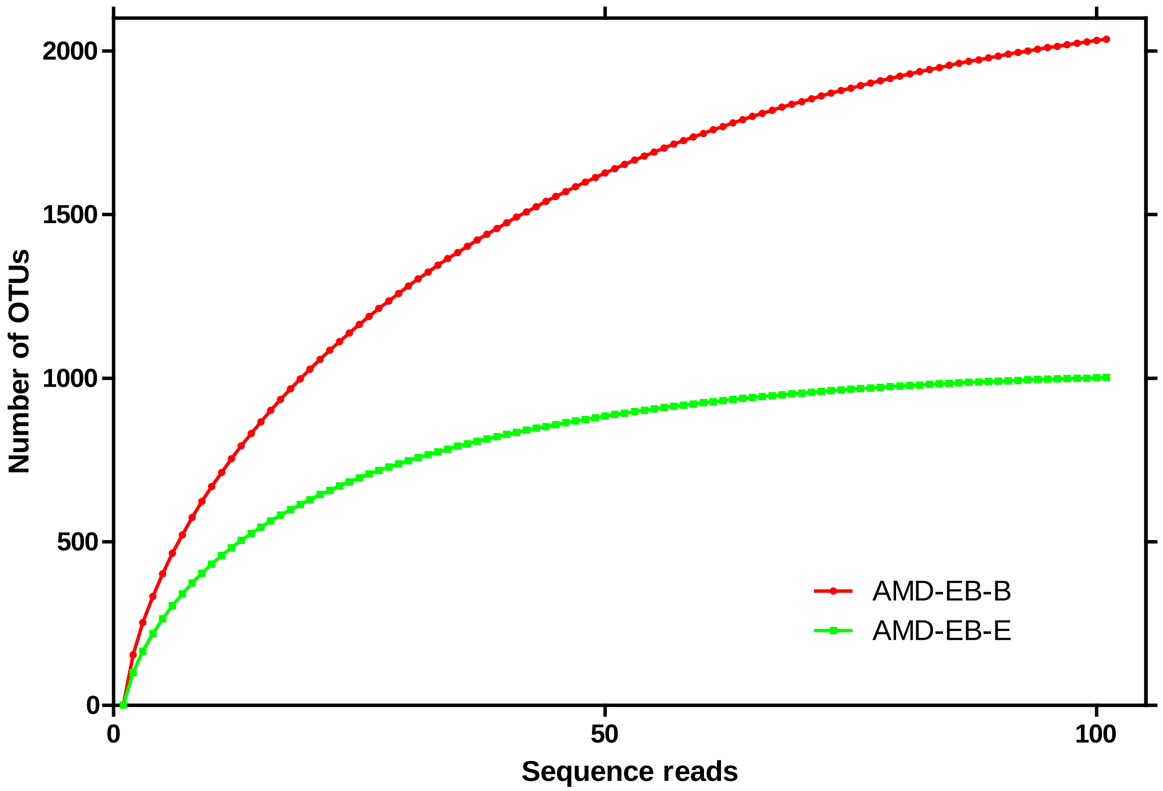

Fig. A.1: Rarefaction curves depicting the rate of increase in the number of OTUs in AMD biofilm of AMD from ‘Onyeama’ coal mine based on UCLUST and CD-HIT clustering. AMD-EB-B represents 16S rRNA gene (red curve), and AMD-EB-E represents ITS (green curve)

Table A.4: Dominant Bacteria OTUs in AMD biofilm water from Onyeama coal mine whose relative abundance is at least 1% of the total valid sequence reads

OTUs	Ratio (%)	Count
*Paludibacter_uc**	4.1	1081
*HE804166_s***	4.1	1072
Rubrivivax gelatinosus group	3.8	996
Novosphingobium flavum group	2.9	765
Pseudomonas fulva group	2.6	680
*Holophaga_uc**	2.4	638
Geothrix fermentans	1.9	493
Undibacterium macrobrachii	1.9	488
Undibacterium danionis	1.8	474
*HE604089_s***	1.6	412
Novosphingobium bradum	1.4	372
*GQ052695_s***	1.3	353
Thauera selenatis group	1.3	351
*AJ488070_s***	1.1	277
Dechloromonas denitrificans group	1.0	264

***uncultured class OTUs**

**** Tentative species of the type bacterial strain with the corresponding GenBank accession number**

Table A.5: Dominant Eukarya OTUs in AMD biofilm water from Onyeama coal mine whose relative abundance is at least 0.5% of the total valid sequence reads

Fungi			Animalia			Plantae		
**OTUs**	**Ratio**	**Count**	**OTUs**	**Ratio**	**Count**	**OTUs**	**Ratio**	**Count**
Aspergillaceae sp_JN839953	30.4	12263	*Ciliophora_f_uc_s*	9.3	3759	*Chlorophyta_f_uc_s*	3.5	1421
Ascomycota sp_KT758121	17.1	6889	*Arthropoda_o_uc_s*	8.3	3371	*Chlorophyta_o_uc_s*	1.9	787
Aspergillus halophilicus	1.5	599	*Ciliophora_o_uc_s*	3.0	1216	*Trebouxiales_uc_s*	0.5	206
GS23_uc_s	1.1	441						
Fungi_f_uc_s	0.9	370						
Hypocreales_uc_s	0.8	314						
Fungi sp_KF675434	0.8	313						

Table A.6: Taxonomy of bacteria consortium involved in toxic metal sequestrations of AMD

Phylum	Class	Family	OTUs	Bacteria count	
				**At inoculation**	**Post incubation**
Proteobacteria	ϒ-Proteobacteria	Moraxellaceae	*Acinetobacter pittii* group	5	16543
Firmicutes	Bacilli	Planococcaceae	*Sporosarcina koreensis* group	6	2758
Proteobacteria	ϒ-Proteobacteria	Enterobacteriaceae	Enterobacteriaceae group	10	1413
Firmicutes	Bacilli	Bacillaceae	*Bacillus cereus* group	8	1222
Firmicutes	Bacilli	Exiguobacteriaceae	*Exiguobacterium aurantiacum* group	1	1091
Proteobacteria	ϒ-Proteobacteria	Enterobacteriaceae	*FWNZ_s*	1	750
Proteobacteria	ϒ-Proteobacteria	Pseudomonadaceae	*Pseudomonas citronellolis* group	5	683

Supplementary Calculations:

Computation of pollution indices and ecological risk assessments

Contamination factor (CF): determines addition of heavy metals (HMs) in the environment in relation to contents of the metals in unpolluted freshwater as the background values (Hakanson, 1980). It is calculated as:

$$CF = \frac{Cn}{Bn} \quad \ldots\ldots\ldots\ldots\ldots\ldots\ldots\ldots \quad (1)$$

where C_n is the concentration of the metal n and B_n is the natural local background concentration of metal n. CF is categorized into four as low contamination, CF < 1; moderate contamination, 1 < CF ≤ 3; high contamination, 3 < CF ≤ 6; and very high contamination, CF > 6.

Enrichment factor (EF): evaluates the enrichment of each metal in environment on basis of linear relationship between the HMs found in the study environment with those of reference or background (unpolluted) environment (Lu et al., 2014). According to Kumar et al. (2018), Fe was taken as conservative tracer in finding the degree of alterations of natural from human activities since Fe is the key sorbet phase for trace elements. It is calculated based on equations of Muller (1969) as:

$$EF = \frac{C_n}{C_{Fe}} \Big/ \frac{B_n}{B_{Fe}} \quad \ldots\ldots\ldots\ldots\ldots\ldots\ldots\ldots \quad (2)$$

Where C_n and B_n were as state earlier in Eq. (1) above, C_{Fe} is the Fe content (mean, 10.2 mg l^{-1}) in a river that receives the AMD and B_{Fe} is the Fe content in background environment. EF is categorized into: no enrichment, EF < 1; less enrichment, 1 ≤ EF < 3; moderate enrichment, 3 ≤ EF < 5; moderately high enrichment, 5 ≤ EF < 10; high enrichment, 10 ≤ EF < 25; very high enrichment, 25 ≤ EF < 50; and exceptionally high enrichment, EF > 50.

Geo-accumulation index (I_{geo}): determines the level of HMs pollution based on HMs contents and it was calculated following Muller (1969) methods as:

$$I_{geo} = log_2\left(\frac{C_n}{K \times B_n}\right) \quad \ldots\ldots\ldots\ldots\ldots\ldots\ldots\ldots \quad (3)$$

where C_n is the concentration of metal n and B_n is as indicated above. The factor K is the background matrix correction factor due to lithospheric effects, which is usually defined as 1.5 according to Muller (1969). It was ranked as: no pollution, $I_{geo} < 0$; moderate pollution, $0 \leq I_{geo} < 1$; strong pollution, $1 \leq I_{geo} < 2$; high pollution, $2 \leq I_{geo} < 3$; very high pollution, $3 \leq I_{geo} < 4$; severe pollution, $4 \leq I_{geo} < 5$; and very severe pollution, $I_{geo} \geq 5$.

Pollution load index (PLI): is the nth root of the multiplication of CF of individual HMs.

$$PLI = (CF_1 \times CF_2 \times CF_3 \times \ldots \times CF_n)^{\frac{1}{n}}, \ldots \qquad (4)$$

CF is the contamination factor as described before, and PLI is categorized as: no pollution, PLI < 0; presence of baseline level of pollutant, 0 < PLI ≤ 1; polluted system, 1 < PLI ≤ 10; highly polluted system, 10 < PLI ≤ 100; progressively deteriorating environment PLI > 100.

Pollution index (PI): involved using a weighted average based on the average and maximum values of CF to determine the quality of the environmental samples (Nemerow, 1991), and thus calculated as:

$$PI = \sqrt{\frac{(CF_{av})^2 + (CF_{max})^2}{2}} \ldots \qquad (5)$$

CF_{av} and CF_{max} are the average and maximum values of CF, respectively. It is classified as: no pollution, PI < 0.7; slight pollution, 0.7 < PI ≤ 1; moderate pollution, 1 < PI ≤ 2; high pollution, 2 < PI ≤ 3; and severe pollution, PI > 3.

Modified pollution index (MPI): is based on enrichment factor of the environmental stressor and calculated as:

$$MPI = \sqrt{\frac{(EF_{av})^2 + (EF_{max})^2}{2}} \quad \ldots\ldots\ldots\ldots\ldots\ldots\ldots\ldots\ldots\ldots\ldots\ldots\ldots\ldots\ldots\ldots\ldots \quad (6)$$

EF_{av} and EF_{max} are the average and maximum values of EF, respectively. It is classified as: no pollution, MPI < 1; slightly polluted, 1 < MPI ≤ 2; moderately polluted, 2 < MPI ≤ 3; moderately heavily polluted, 3 < MPI ≤ 5; highly polluted, 5 < MPI ≤ 10; and severely polluted, MPI > 10.

Degree of contamination (C_d): measures the extent to which HMs get introduced to an environment, and it is calculated as:

$$C_d = \sum_{i=1}^{n} CF \quad \ldots\ldots\ldots\ldots\ldots\ldots\ldots\ldots\ldots\ldots\ldots\ldots\ldots\ldots\ldots\ldots\ldots\ldots$$

(7)

Modified degree of contamination (MC_d): measures the extent to which HMs get introduced to an environment, and it is calculated as:

$$C_d = \sum_{i=1}^{n} EF \quad \ldots\ldots\ldots\ldots\ldots\ldots\ldots\ldots\ldots\ldots\ldots\ldots\ldots\ldots\ldots\ldots\ldots\ldots$$

(8)

Ecological risk assessment

Potential ecological risk factor (Er): is based on factor at which CF relates to toxicological response factor (T_r^i) of HMs.

$$E_r^i = T_r^i . CF \quad \ldots\ldots\ldots\ldots\ldots\ldots\ldots\ldots\ldots\ldots\ldots\ldots\ldots\ldots\ldots\ldots\ldots\ldots \quad (9)$$

where T_r^i is the toxic response factor for a given substance (see table A.1). Categories of risk assessment are as: low risk, Er < 40; moderate risk, 40 < Er ≤ 80; considerate risk, 80 < Er ≤ 160; high risk, 160 < Er ≤ 320; and very high risk, Er > 320 of HMs.

Modified potential ecological risk factor (MEr): is based on factor at which EF relates to toxicological response factor (T_r^i) of HMs.

$$ME_r^i = T_r^i . EF \quad \text{...} \quad (10)$$

where T_r^i is as defined above. Categories of risk assessment are as: low risk, MEr < 40; moderate risk, 40 < MEr ≤ 80; considerate risk, 80 < MEr ≤ 160; high risk, 160 < MEr ≤ 320; and very high risk, MEr > 320 of HMs.

Potential ecological risk index (RI): is the summation of all risk factors for HMs in the environment (Hakanson, 1980) and it is calculated as:

$$RI = \sum_i^m E_r^i, \quad \text{...} \quad (11)$$

Categories of risk assessment are as: low risk, RI < 40; moderate risk, 40 < RI ≤ 80; considerate risk, 80 < RI ≤ 160; high risk, 160 < RI ≤ 320; and very high risk, RI > 320 of HMs.

Modified potential ecological risk index (MRI): is the summation of all modified risk factors for HMs in the environment, and it is calculated as:

$$MRI = \sum_i^m ME_r^i, \quad \text{...} \quad (12)$$

Categories of risk assessment are as: low risk, MRI < 40; moderate risk, 40 < MRI ≤ 80; considerate risk, 80 < MRI ≤ 160; high risk, 160 < MRI ≤ 320; and very high risk, MRI > 320 of HMs.

Risk quotient (RQ): is the calculated index of ecological risk assessment based ratio of measured environmental concentration to predicted no-effect concentration (PNEC). In the

present study, PNEC was based on function of permissible limit of HMs in drinking water and the total organic carbon in the AMD. RQ was therefore calculated as:

$$RQ = \frac{C_e}{C_{pl} \times F_{oc}} \ldots\ldots\ldots\ldots\ldots\ldots\ldots\ldots\ldots\ldots\ldots\ldots\ldots\ldots\ldots\ldots\ldots\ldots\ldots\ldots \quad (13)$$

where C_e (mg/l) is the environmental concentration of HMs, C_{pl} is the permissible limit of HMs in surface waters that poses no danger to physiologies of living things, and F_{oc} is the mean organic carbon in the AMD. A RQ ≥ 1 shows the risk posed by toxicants is high but the risk is low if RQ < 1.

Table S.1: Geochemical background values and determination of toxic response values of metal(loid)s

Metal(loid)s	B_n (µg l^{-1})	St^i - value	BPI	Tr^i value
Cd	0.032	30	30	$30 \times \frac{\sqrt{5}}{\sqrt{BPI}} = 12.25$
As	2.0	10	10	10
Pb	3.0	5	5	$5 \times \frac{\sqrt{5}}{\sqrt{BPI}} = 5.0$
Cr	1.0	2	2	$2 \times \frac{\sqrt{5}}{\sqrt{BPI}} = 3.16$
Fe	100	2[a]	2[b]	$2 \times \frac{\sqrt{5}}{\sqrt{BPI}} = 3.16$
Ni	1.5	2[a]	2[b]	$2 \times \frac{\sqrt{5}}{\sqrt{BPI}} = 3.16$
Co	0.1	2[a]	2[b]	$2 \times \frac{\sqrt{5}}{\sqrt{BPI}} = 3.16$

B_n = the geochemical background values of metals in unpolluted freshwater spring within the geographical area of the mine; BPI = bioproduction index; St^i = sedimentological-toxicological factors; Tr^i = toxic-response factor for given metal(loid)

[a] St^i – value of Cr were adopted since there is no standard value available for Fe, Ni and Co.

[b] BPI of Cr were adopted since there is no standard value available for Fe, Ni and Co.

Reference

Muller, G. Index of geo-accumulation in sediments of the Rhine River. *Geol. J.* **2,** 108-118 (1969).

Method A.1: Process leading to Illumina MiSeq sequencing

PCR amplification

1st PCR (Amplicon PCR)

1) Primer information

All primers follow the composition of “Nextera concensus - Sequencing adaptor - Target sequence”.

Bacteria (16S rRNA gene, V3-V4 region)

**Forward 1st_341F**	TCGTCGGCAGCGTC-AGATGTGTATAAGAGACAG-CCTACGGGNGGCWGCAG (50mer)
**Reverse 1st_805R**	GTCTCGTGGGCTCGG-AGATGTGTATAAGAGACAG-GACTACHVGGGTATCTAATCC (55mer)

Eukarya (ITS2 region)

**Forward ITS3-Mi**	TCGTCGGCAGCGTC-AGATGTGTATAAGAGACAG-GCATCGATGAAGAACGCAGC (53mer)
**Reverse ITS4-Mi**	GTCTCGTGGGCTCGG-AGATGTGTATAAGAGACAG-TCCTCCGCTTATTGATATGC (54mer)

PCR reagents and volume

Reagent	Volume
**10X buffer**	2.5 μl
**dNTP**	2.5 μl
**Forward Primer** (10 pmole/μl)	1 μl
**Reverse Primer** (10 pmole/μl)	1 μl
**Taq polymerase** (TaKaRa Ex Taq DNA polymerase, 1000 U)	0.25 μl
**DNA**	2 μl
**D.W.**	15.75 μl

**Total**	25 µl

PCR condition

Step	°C	Min	Cycle
Initial denaturation	95	3:00	
Denaturation	95	0:30	25 cycles
Annealing	55	0:30	
Extension	72	0:30	
Final extension	72	5:00	
Hold	4	∞	

2nd PCR (Index PCR)

1) PCR primer

**Forward Index i5**	AATGATACGGCGACCACCGAGATCTACAC-XXXXXXXX-TCGTCGGCAGCGTC (51mer)<br>(composition : Left – i5 (XXXXXXXX) – Right)
**Reverse Index i7**	CAAGCAGAAGACGGCATACGAGAT-XXXXXXX-GTCTCGTGGGCTCGG (47mer)<br>(composition : Left – i7 (XXXXXXXX) – Right)

* Index sequence table

Index 1 (i7)	Sequence	Index 2 (i5)	Sequence
N701	TCGCCTTA	S502	CTCTCTAT
N702	CTAGTACG	S503	TATCCTCT
N703	TTCTGCCT	S505	GTAAGGAG
N704	GCTCAGGA	S506	ACTGCATA

N705	AGGAGTCC	S507	AAGGAGTA
N706	CATGCCTA	S508	CTAAGCCT
N707	GTAGAGAG	S510	CGTCTAAT
N710	CAGCCTCG	S511	TCTCTCCG
N711	TGCCTCTT	S513	TCGACTAG
N712	TCCTCTAC	S515	TTCTAGCT
N714	TCATGAGC	S516	CCTAGAGT
N715	CCTGAGAT	S517	GCGTAAGA
N716	TAGCGAGT	S518	CTATTAAG
N718	GTAGCTCC	S520	AAGGCTAT
N719	TACTACGC	S521	GAGCCTTA
N720	AGGCTCCG	S522	TTATGCGA
N721	GCAGCGTA		
N722	CTGCGCAT		
N723	GAGCGCTA		
N724	CGCTCAGT		
N726	GTCTTAGG		
N727	ACTGATCG		
N728	TAGCTGCA		
N729	GACGTCGA		

PCR reagents and volume

Reagent	Volume
**10X buffer**	2.5 µl
**dNTP**	2.5 µl

**Forward Primer** (10 pmole/µl)	1 µl
**Reverse Primer** (10 pmole/µl)	1 µl
**Taq polymerase** (TaKaRa Ex Taq DNA polymerase, 1000 U)	0.25 µl
**DNA** (1st PCR Product)	2 µl
**D.W.**	15.75 µl
**Total**	25 µl

PCR condition

Step	°C	Min	Cycle
Initial denaturation	95	3:00	
Denaturation	95	0:30	8 cycles
Annealing	55	0:30	
Extension	72	0:30	
Final extension	72	5:00	
Hold	4	∞	

Library preparation and Quality check

**Quantity of library**	Invitrogen, Quanti-iT PicoGreen dsDNA Assay Kit

**Quality of library**	Agilent Technologies, US., Agilent 2100 Bioanalyzer System
**Removal of short fragment**	CleanNA, Netherlands, CleanPCR
**Thermal cycler for Quantitative PCR**	Bio-Rad, CFX 96 Real-Time system (C1000)
**Reagents for Quantitative PCR**	KAPA Biosystems, KAPA SYBR FAST Universal 2X qPCR Master KAPA Biosystems, Library Quantification DNA Standards only-Illumina

Sequencing

**Sequencing platform**	Illumina, MiSeq
**Sequencing kit**	Illumina, MiSeq Reagent Kit v2 (500-cycles)

Table A.7: Heavy metals/metalloids cocktails used for growth kinetics and urease activity study

Metal cocktails	Cd	Pb	Co	Ni	As
Low concentrations (mg l^{-1})	27.9	118.7	16.2	16.2	61.5
Medium concentrations (mg l^{-1})	55.7	237.3	32.4	32.3	123.1
High concentrations (mg l^{-1})	139.3	593.3	81.1	80.7	307.6
